# A randomised controlled trial of in-patient treatment for anorexia nervosa in medically unstable adolescents

**DOI:** 10.1186/2050-2974-3-S1-O44

**Published:** 2015-11-23

**Authors:** Sloane Madden, Jane Miskovic-Wheatley, Andrew Wallis, Michael Kohn, James Lock, Daniel LeGrange, Booil Jo, Simon Clarke, Paul Rhodes, Phillipa Hay, Stephen Touyz

**Affiliations:** 1 The Sydney Children's Hospital Network, Australia; 2 The University of Sydney, Australia; 3 Stanford University, USA; 4 Univeristy of Chicago, USA; 5 Westmead Hospital, Australia; 6 University of Western Sydney, Australia

## Background

Anorexia Nervosa (AN) is a serious disorder, with high costs due to hospitalisation. International treatments vary with prolonged hospitalisations in Europe and shorter hospitalisations in the USA. Uncontrolled studies suggest longer initial hospitalisations that normalise weight produce better outcomes and less admissions than shorter hospitalisations with lower discharge weights.

## Methods

A randomised controlled trial of 82 adolescents, with DSM-IV AN and medical instability comparing brief hospitalisation for medical stabilisation (MS) and hospitalisation for weight restoration (WR) to 90% expected body weight (EBW) (1:1), both followed by 20 sessions of manualised, family based treatment (FBT).

## Results

Primary outcome was hospital days, following initial admission, at 12-month follow-up. Secondary outcomes were total hospital days to 12-months and full remission (EBW>95% and global Eating Disorder Examination score within 1 SD of published means). There was no significant difference between groups in hospital days used following initial admission. There were significantly more total hospital days used and post-protocol FBT sessions in the WR group. There were no moderators of primary outcome, but participants with higher eating psychopathology and compulsive features reported better outcomes in the MS group.

## Conclusions

Outcomes are similar with hospitalisations for MS or WR when combined with FBT. Cost savings would result from combining shorter hospitalisation with FBT.

